# Educational Gaps in Dermatologic Diagnoses Among Otolaryngology Residents

**DOI:** 10.1002/oto2.70017

**Published:** 2024-09-23

**Authors:** Sasan Darain Noveir, Wasiq Nadeem, Carol E. Cheng, Matthew K. Lee

**Affiliations:** ^1^ David Geffen School of Medicine at University of California Los Angeles Los Angeles California USA; ^2^ Division of Otolaryngology–Head and Neck Surgery Cedars‐Sinai Medical Center Los Angeles California USA; ^3^ Department of Medicine, Division of Dermatology David Geffen School of Medicine at University of California Los Angeles Los Angeles California USA

**Keywords:** dermatology, diagnostic assessment, otolaryngology, resident curriculum

## Abstract

Otolaryngologists frequently serve as the first touchpoint for patients presenting with dermatologic conditions of the head and neck. This study aims to identify and quantify gaps in dermatologic training among otolaryngology residents, and to assess their diagnostic accuracy in comparison to dermatology residents. It comprised 14 multiple‐choice questions focused on common dermatologic diagnoses related to the head and neck. Sixty‐one dermatology and 36 otolaryngology residents participated in the study. Dermatology residents significantly outperformed otolaryngology residents, with average scores of 90% (SD = 8) compared to 71% (SD = 10) (*P* < .001). The observed effect size (Cohen's *d* = 2.010) significantly exceeded the expected effect size (0.603). Otolaryngology residents performed significantly lower on 7 out of the 14 questions. Analysis based on postgraduate year level showed no significant differences in scores within dermatology (*P* = .119) or otolaryngology (*P* = .402) residency programs.

Otolaryngologists frequently serve as the first touchpoint for patients presenting with dermatologic conditions of the head and neck. Data from the National Ambulatory Medical Care Survey indicates that otolaryngologists managed approximately 7,510,000 skin‐related visits between 2001 to 2010.^1^ Studies have demonstrated existing gaps in the ability of nondermatology physicians to accurately diagnose common dermatologic conditions.^2^, ^3^ Specifically in otolaryngology, a recent survey of senior otolaryngology residents and pediatric otolaryngology fellows demonstrated that a large majority (78% of respondents) reported inadequate exposure to dermatologic vascular anomalies during their training.^4^ Moreover, all respondents agreed that enhanced training in these areas would benefit patient care.^4^ There is a currently lack of empiric data on whether otolaryngology training adequately prepares its trainees in the diagnosis and management of dermatologic conditions in the head and neck. This study aims to identify the potential gaps in dermatologic training among otolaryngology residents, thereby highlighting areas for curriculum development and enhancement within the field.

## Methods

The survey was emailed to all otolaryngology and dermatology residents across the United States via their program directors and program coordinators. Participation in the survey was voluntary and anonymous.

The survey was designed to assess the diagnostic abilities of residents regarding common dermatologic conditions affecting the head and neck. It was developed collaboratively by an otolaryngology program director and a dermatology fellowship director to ensure clinical relevance and comprehensive coverage of the diagnostic skills required by otolaryngology residents. The survey consisted of 14 multiple‐choice questions, each featuring a representative image of a common skin condition that otolaryngologists would likely encounter in a clinical setting (see Supplemental File, available online). Images were used with permission from VisualDx.^5^

Data analysis included independent *t* tests to compare total correct scores and individual correct answer choices between otolaryngology and dermatology residents. Analysis of variance was utilized to assess intra‐group variances across different postgraduate years (PGYs). A post hoc power analysis, with 80% power and an *α* level of .05, was conducted to determine expected effect size, while Cohen's *d* was used to calculate the observed effect size. This study was deemed exempt by the Institutional Review Boards of Cedars‐Sinai Medical Center and University of California Los Angeles.

## Results

Sixty‐one dermatology and 36 otolaryngology residents participated in the study. Among the dermatology respondents, 41% were second‐year residents (PGY‐2), 26% were third‐year (PGY‐3), and 33% were fourth‐year (PGY‐4). For the otolaryngology respondents, 33% were first‐year residents (PGY‐1), 22% were PGY‐2, 17% were PGY‐3, 25% were PGY‐4, and 3% were sixth‐year (PGY‐6).

Dermatology residents achieved a mean score of 90% (SD = 8), significantly higher than the mean score of 71% (SD = 10) for otolaryngology residents (*P* < .001). The expected effect size was calculated to be 0.603, while the observed effect size analysis (Cohen's *d*) yielded a value of 2.010. Analysis based on PGY level showed no significant differences in scores within dermatology (*P* = .119) or otolaryngology (*P* = .402) residency programs. When analyzing individual survey questions, dermatology residents outperformed otolaryngology residents on 7 questions, while otolaryngology residents scored higher on 1 question (Table 1). Notably, otolaryngology residents more accurately identified milia compared to dermatology residents (*P* = .019). For this question, the most common incorrect response for otolaryngology residents was periorificial dermatitis, comprising 67% of their incorrect answers (n = 6), whereas dermatologists incorrectly chose syringoma, accounting for 96% of their incorrect responses (n = 24).

**Table 1:** Percentage of Correct Responses Among Dermatology and Otolaryngology Residents

Question	Correct answer	Dermatology, %	Otolaryngology, %	*P* value
1	Sebaceous hyperplasia	98	33	<.001*
2	Keloid	98	92	.111
3	Rhinophyma	100	100	1
4	Actinic keratosis	67	39	.006*
5	Seborrheic keratosis	97	28	<.001*
6	Basal cell carcinoma	98	81	.002*
7	Melanoma	100	97	0.195
8	Epidermoid cyst	95	72	.001*
9	Squamous cell carcinoma	82	81	0.865
10	Acrochordon	100	83	.001*
11	Impetigo	92	97	0.289
12	Verruca vulgaris	75	19	<.001*
13	Milia	61	83	.019*
14	Infantile hemangioma	92	86	0.378
Total		90	71	<.001*

* Indicate statistically significant.

## Discussion

This study identifies a significant deficiency in dermatologic diagnostic accuracy among otolaryngology residents when compared to their counterparts in dermatology. A post hoc power analysis validated the significant difference in scores, confirming that the study had adequate statistical power. Otolaryngology residents' lower performance on 7 specific questions indicates targeted opportunities to enhance the educational content.

Interestingly, although cutaneous malignancies are more emphasized in otolaryngology training, basal cell carcinoma was a diagnostic challenge for otolaryngology residents, suggesting the need for more comprehensive training in common skin cancers. Notably, otolaryngology residents diagnosed milia more accurately than dermatology residents. In looking more deeply into answer patterns, this was primarily due to dermatology residents confusing it with syringoma, a condition that closely resembles milia but is much less common in otolaryngology practice.

These study findings are consistent with the existing literature that points to a general deficit in dermatologic training among non‐dermatology physicians. A previous report found that primary care physicians initially misdiagnose or fail to diagnose 76% of skin conditions prior to referring patients to a dermatologist.^2^ Another study of pediatricians reflected similar challenges, showing that their diagnostic concordance with dermatologists was only 19.8%.^3^ Given that around half of medical schools require 10 or fewer hours of dermatology instruction, the responsibility for targeted dermatology education largely falls on otolaryngology residency programs.^6^ Previous research has shown the benefits of an online educational intervention, which significantly improved primary care physician's ability to diagnose pigmented lesions.^7^

The limitations of this study include a low response rate in both residencies. Additionally, while the survey was collaboratively developed to ensure clinical relevance, it was not formally validated. Future research should focus on identifying the most common dermatologic conditions encountered by otolaryngologist to target educational enhancements. Additionally, integrating and evaluating dermatologic training modules within otolaryngology residencies would help improve residents' diagnostic accuracy. Finally, with the launch of the Otolaryngology Core Curriculum by the American Academy of Otolaryngology–Head and Neck Surgery (AAO‐HNSF), it would be prudent to re‐evaluate whether this educational gap still exists after completion of the full 2 year curriculum.^8^, ^9^

## Author Contributions

**Sasan Darain Noveir**, data analysis, manuscript preparation, editing, and review; **Wasiq Nadeem**, data analysis, manuscript preparation, editing, and review; **Carol E. Cheng**, conception, design, supervision, manuscript editing; **Matthew K. Lee**, conception, design, supervision, manuscript editing.

## Disclosures

### Competing interests

None.

### Funding source

None.

## Supporting information

Supporting information.
