# Supplementary material for: Educational Gaps in Dermatologic Diagnoses Among Otolaryngology Residents
Source: OTO Open. 2024 Sep 23;8(3):e70017. doi: 10.1002/oto2.70017 (PMC11420511; doi:10.1002/oto2.70017)
Supplement: Supplementary file 1 — Supporting information. [file OTO2-8-e70017-s001.docx]

Supplemental content: Resident Survey

Images used with permission from VisualDx ([www.visualdx.com](http://www.visualdx.com))

For each question, please select the diagnosis that corresponds to the image.

1.
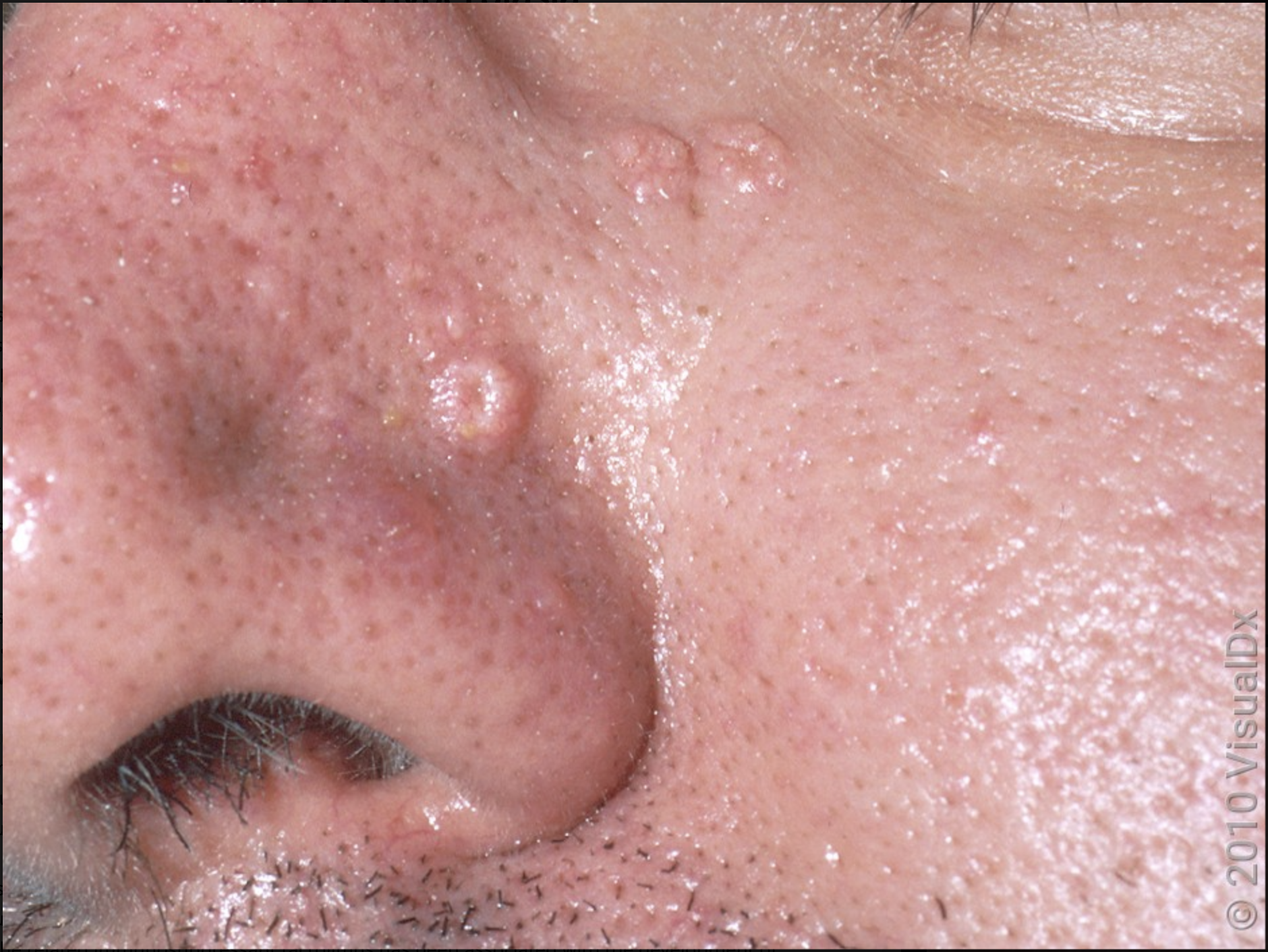

   1. Actinic keratosis
   2. Sebaceous hyperplasia
   3. Basal cell carcinoma
   4. Eruptive xanthoma
   5. Seborrheic dermatitis

**Correct answer:**  b. Sebaceous hyperplasia

1.
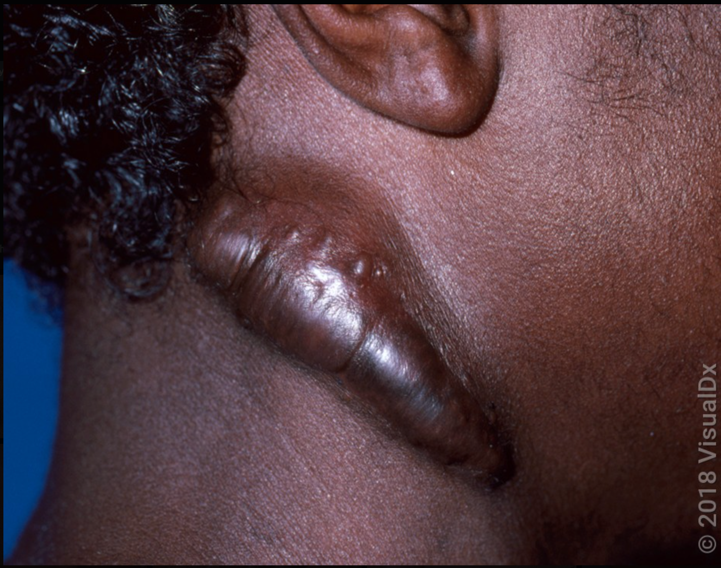

   1. Hypertrophic scar
   2. Lobomycosis
   3. Dermatofibroma
   4. Sarcoidosis
   5. Keloid

**Correct answer:** e. Keloid

1.
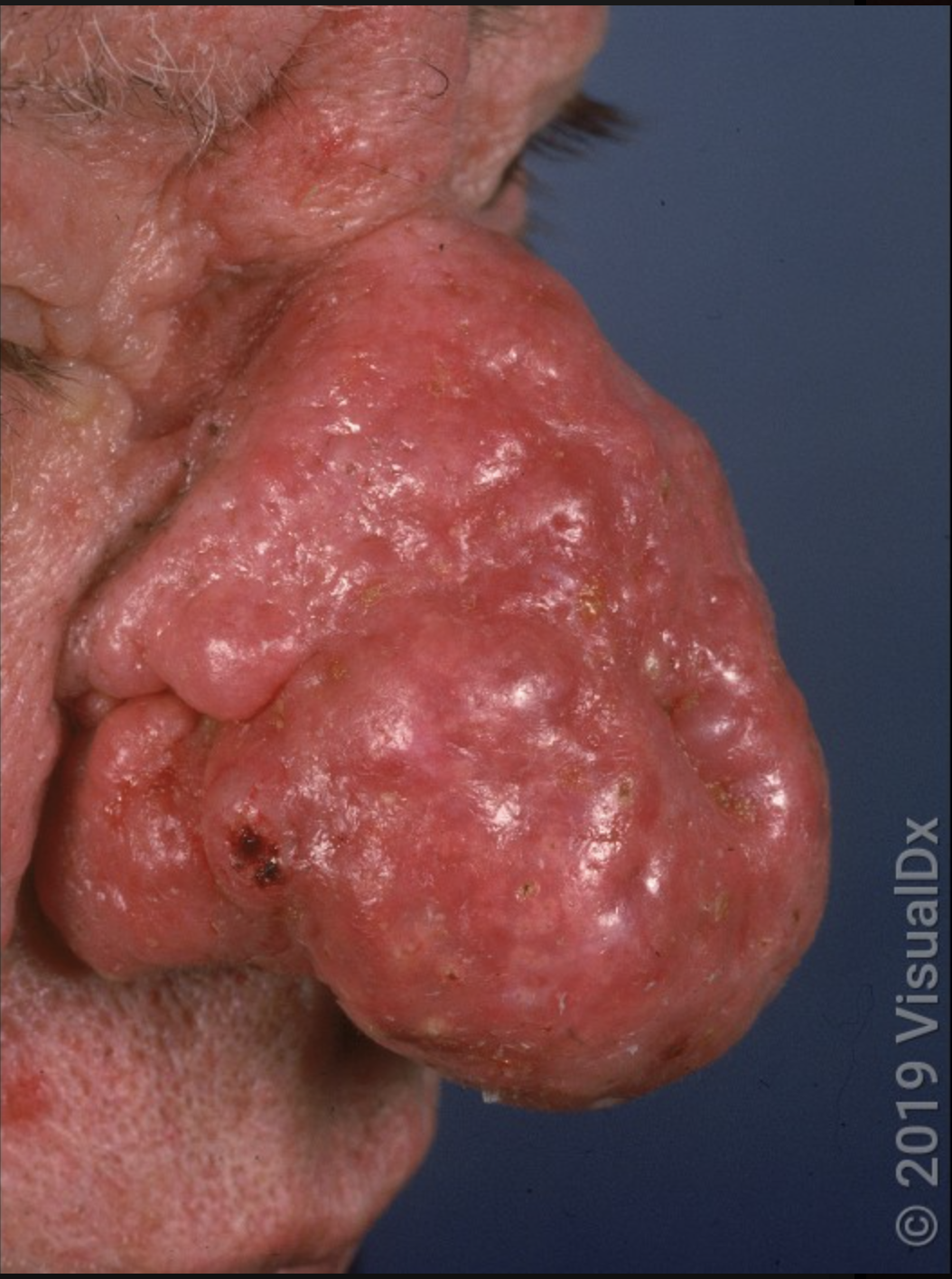

   1. Rhinophyma
   2. Sarcoidosis
   3. Sebaceous adenoma
   4. Acne vulgaris
   5. Cellulitis

**Correct answer:** a. Rhinophyma

1.
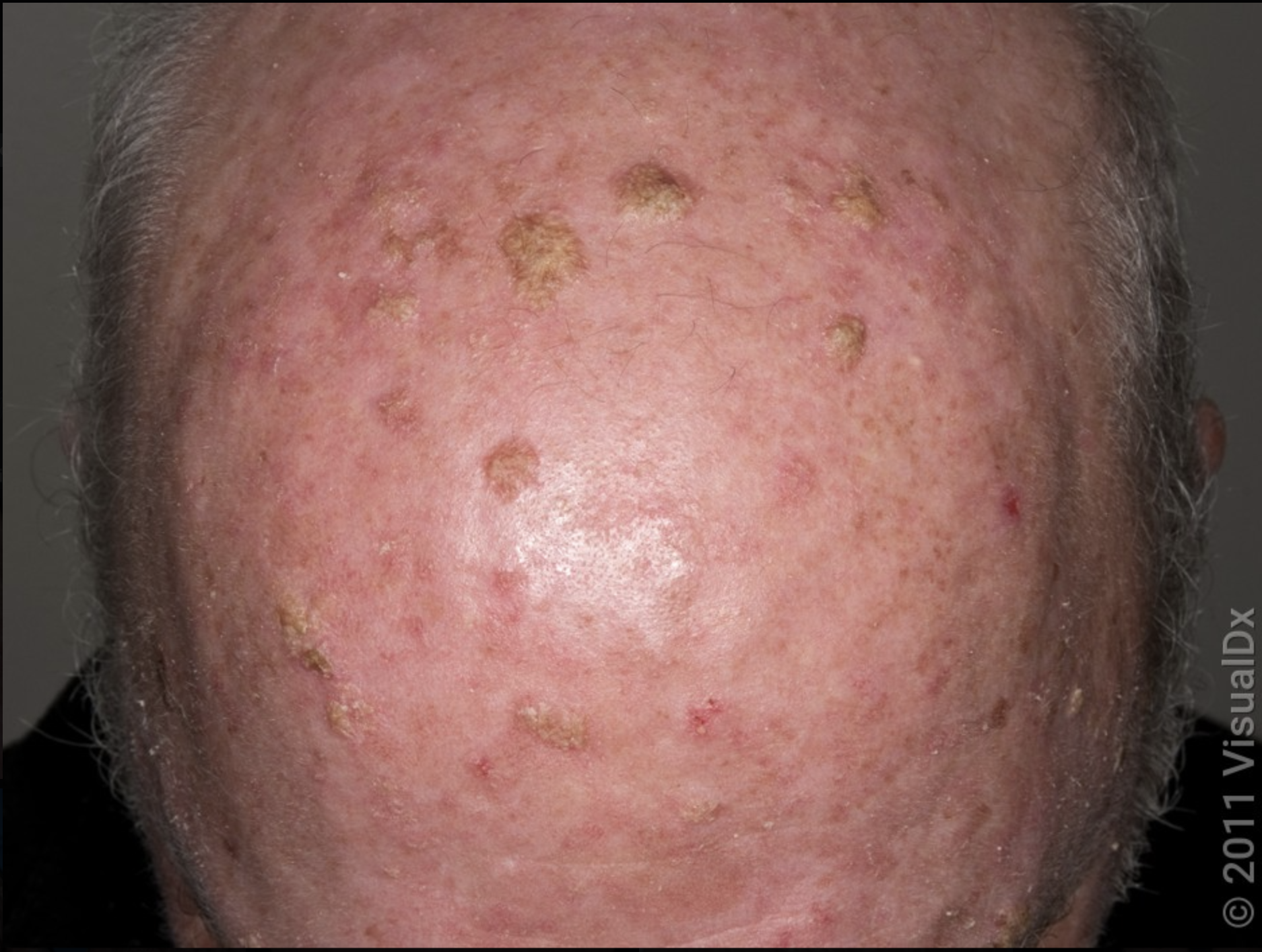

   1. Seborrheic keratosis
   2. Verruca vulgaris
   3. Seborrheic dermatitis
   4. Actinic keratosis
   5. Squamous cell carcinoma

**Correct answer:** d. Actinic keratosis

1.
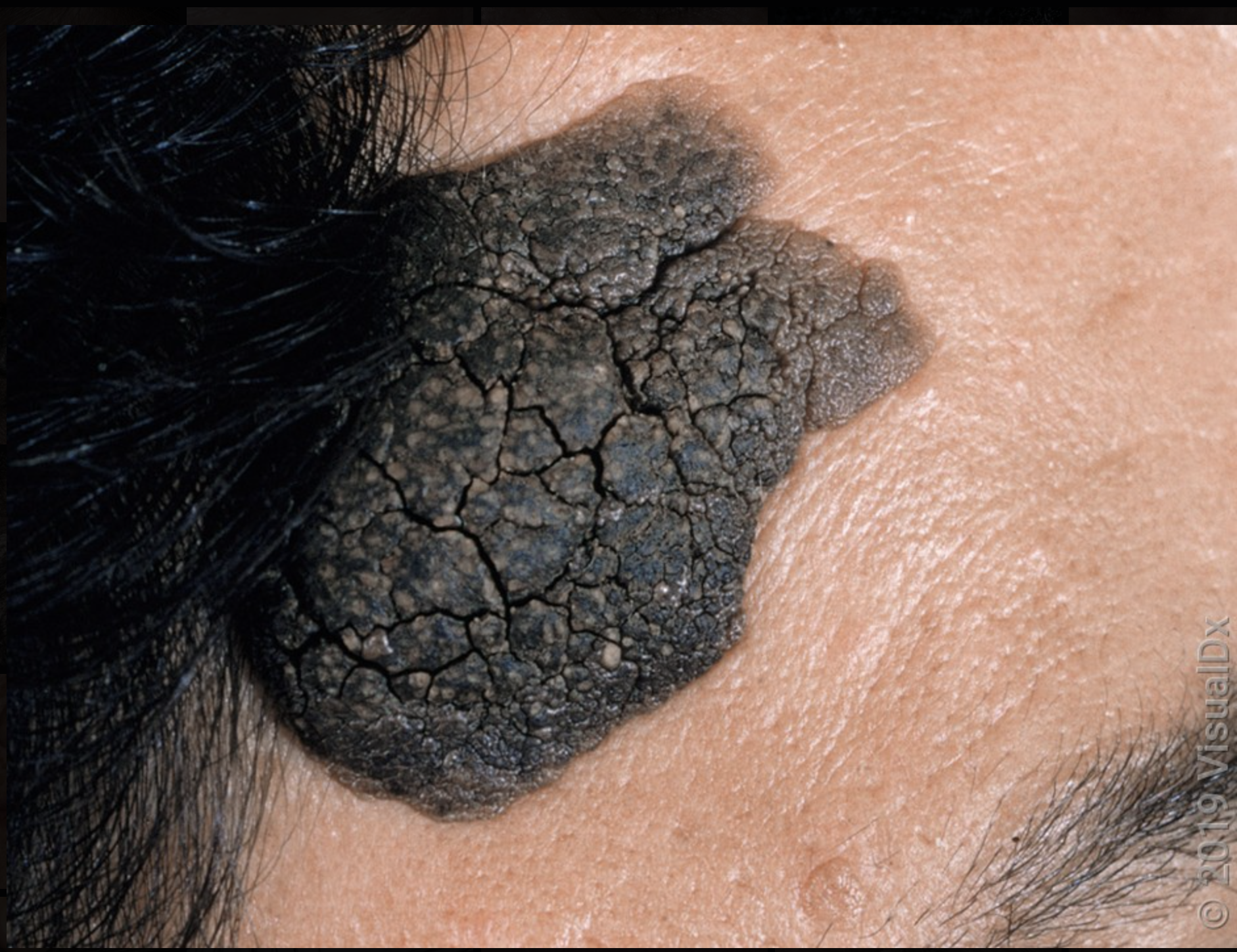

   1. Verruca vulgaris
   2. Melanocytic nevus
   3. Melanoma
   4. Nevus sebaceous
   5. Seborrheic keratosis

**Correct answer:** e. Seborrheic keratosis

1.
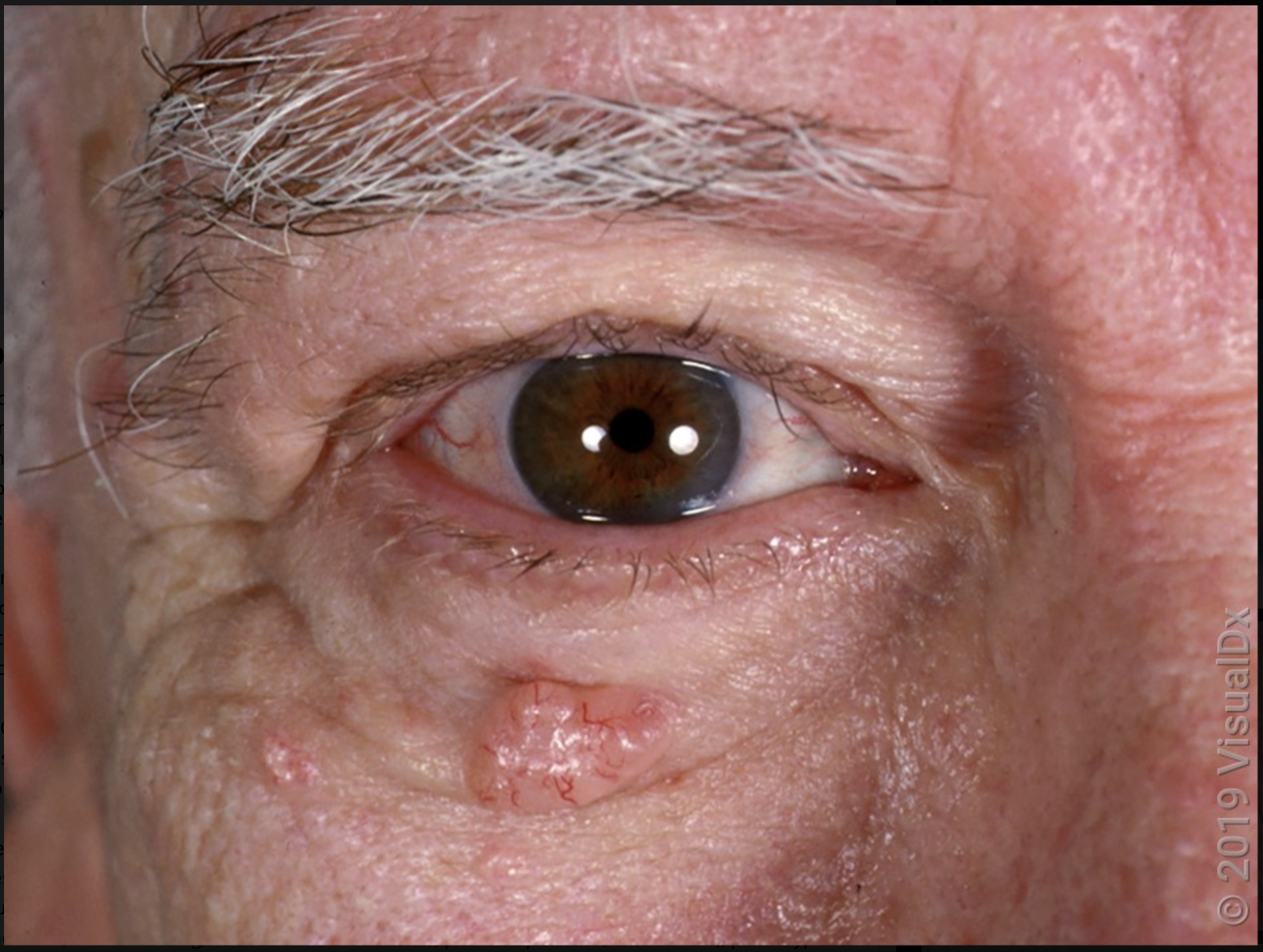

   1. Syringoma
   2. Sebaceous hyperplasia
   3. Keratoacanthoma
   4. Basal cell carcinoma
   5. Intradermal nevus

**Correct answer:** d. Basal cell carcinoma

1.
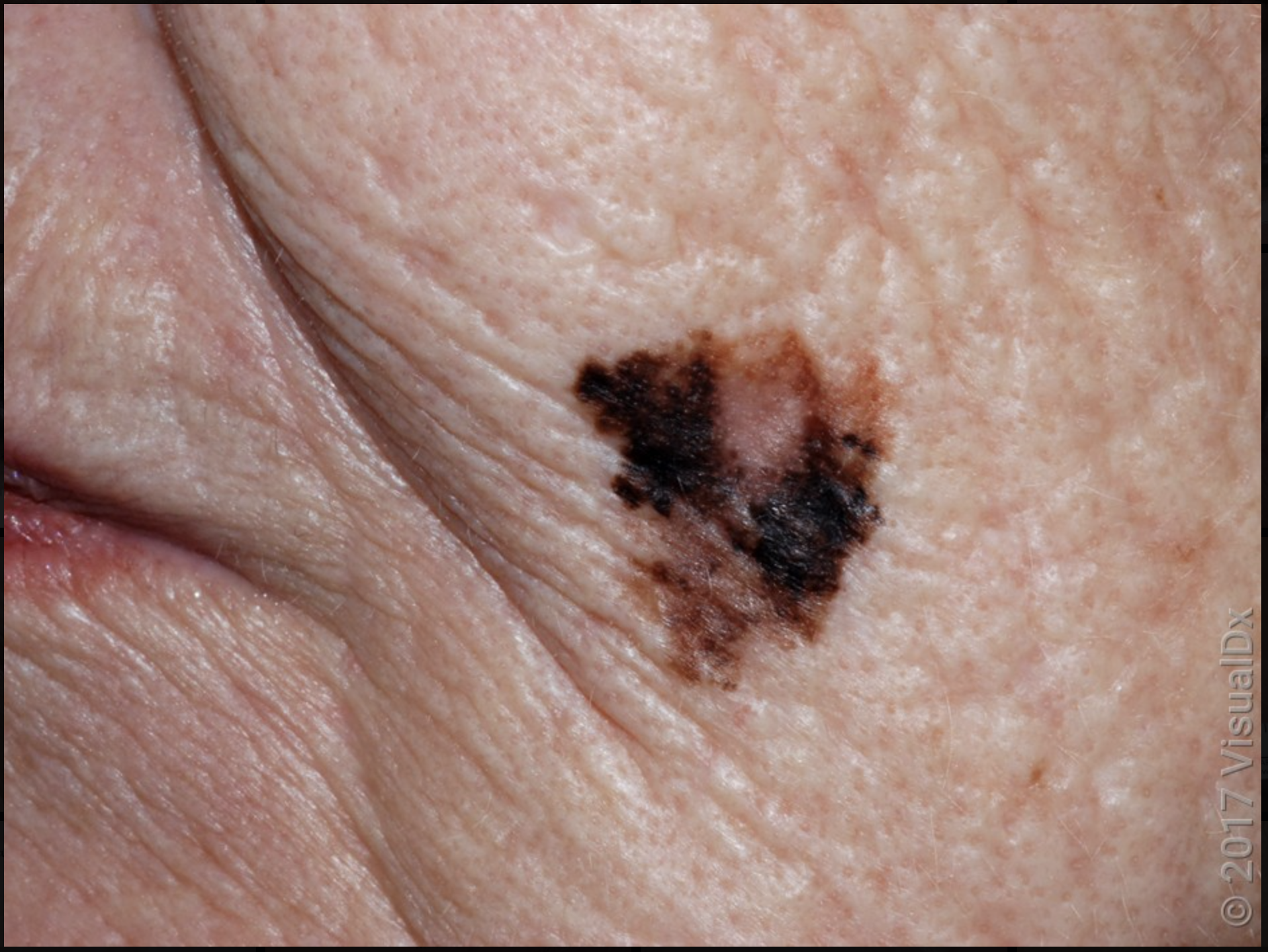

   1. Atypical nevus
   2. Solar lentigo
   3. Melanoma
   4. Seborrheic keratosis
   5. Pigmented basal cell carcinoma

**Correct answer:**  c. Melanoma

1.
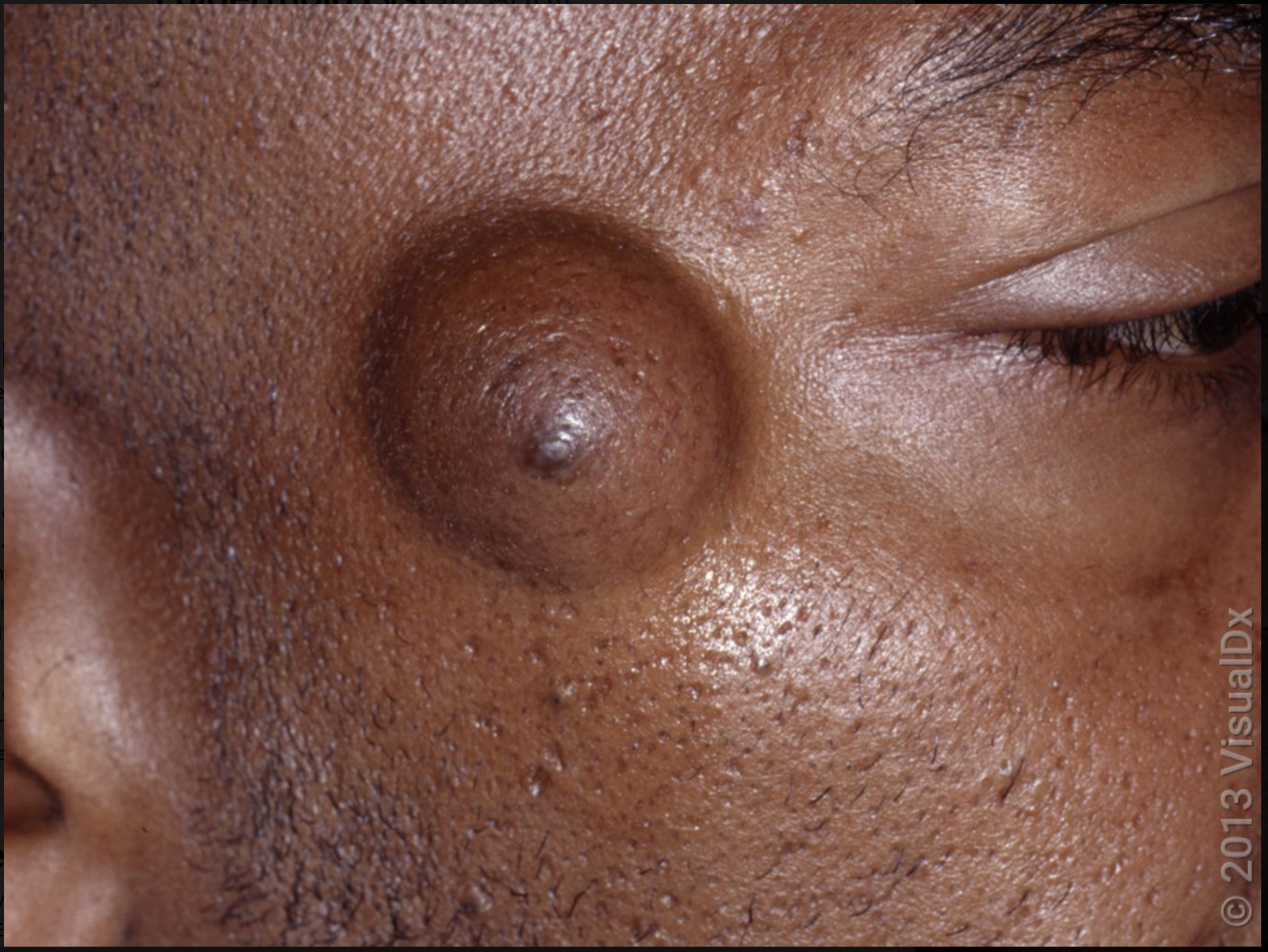

   1. Epidermoid cyst
   2. Dermatofibroma
   3. Abscess
   4. Lipoma
   5. Pilomatrixoma

**Correct answer:** a. Epidermoid cyst

1.
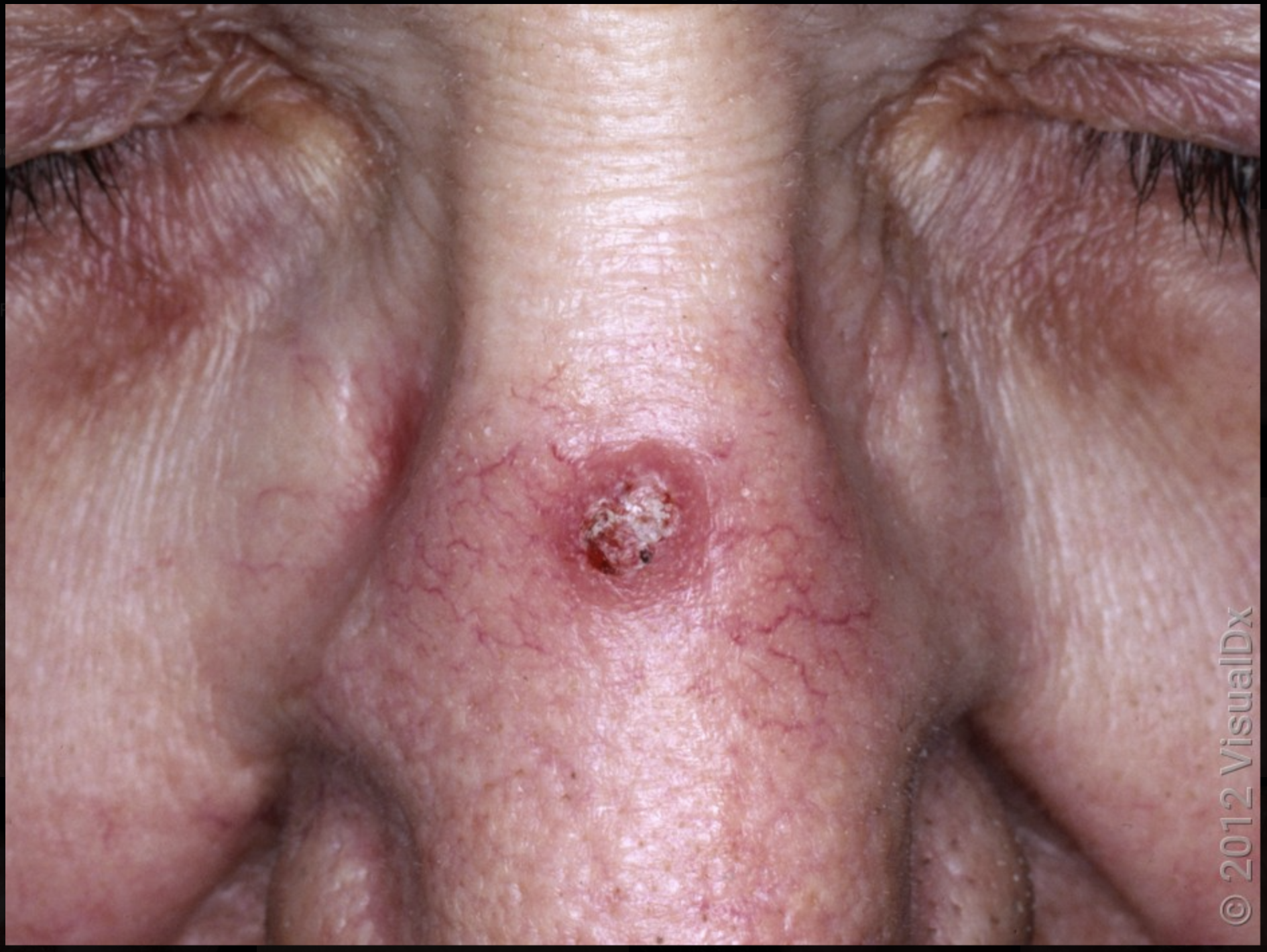

   1. Basal cell carcinoma
   2. Squamous cell carcinoma
   3. Benign lichenoid keratosis
   4. Fibrous papule
   5. Verruca vulgaris

**Correct answer:** b. Squamous cell carcinoma

1.
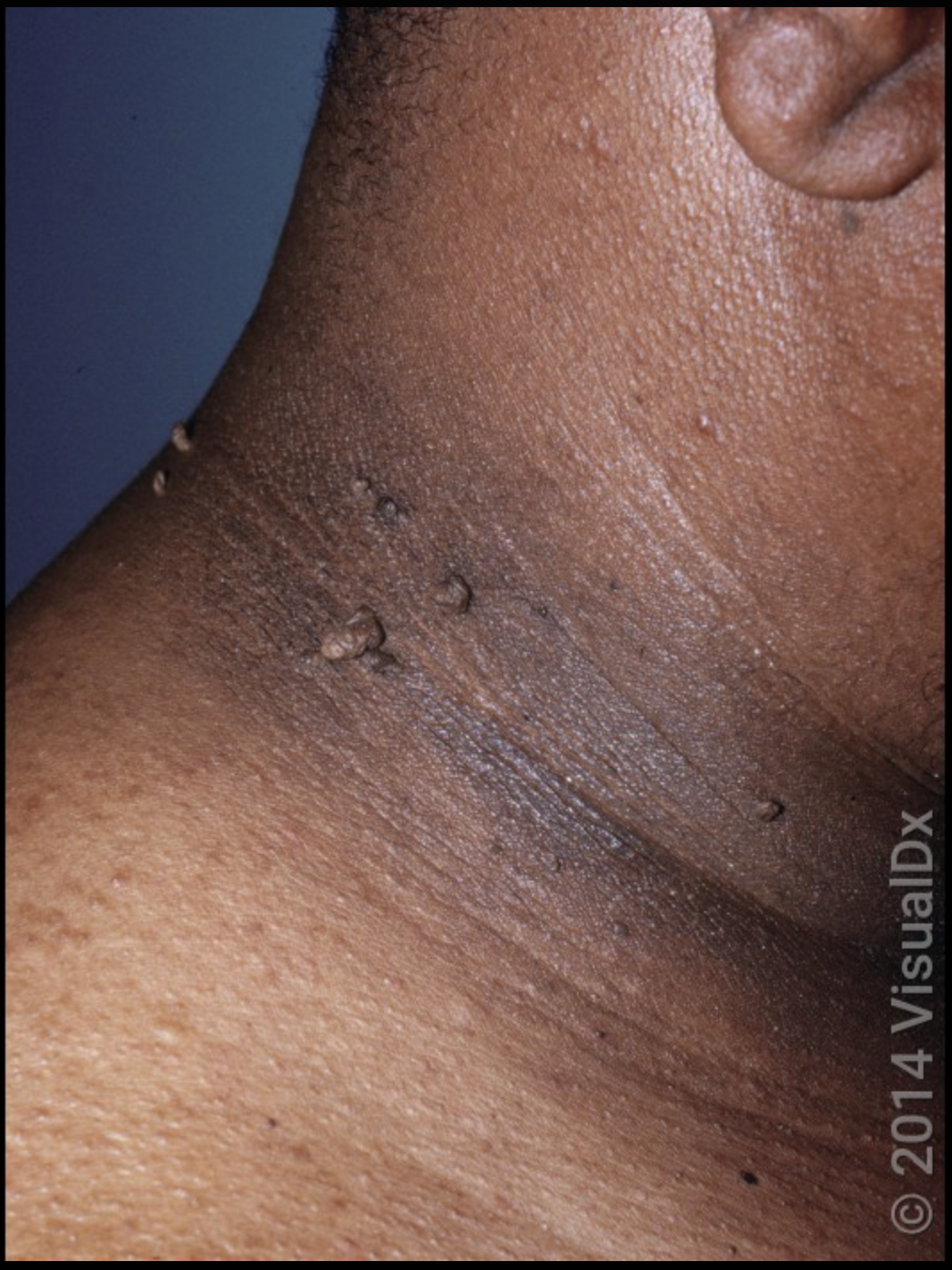

   1. Molluscum contagiosum
   2. Seborrheic keratosis
   3. Acrochordon
   4. Verruca vulgaris
   5. Neurofibromas

**Correct answer:** c. Acrochordon

1.
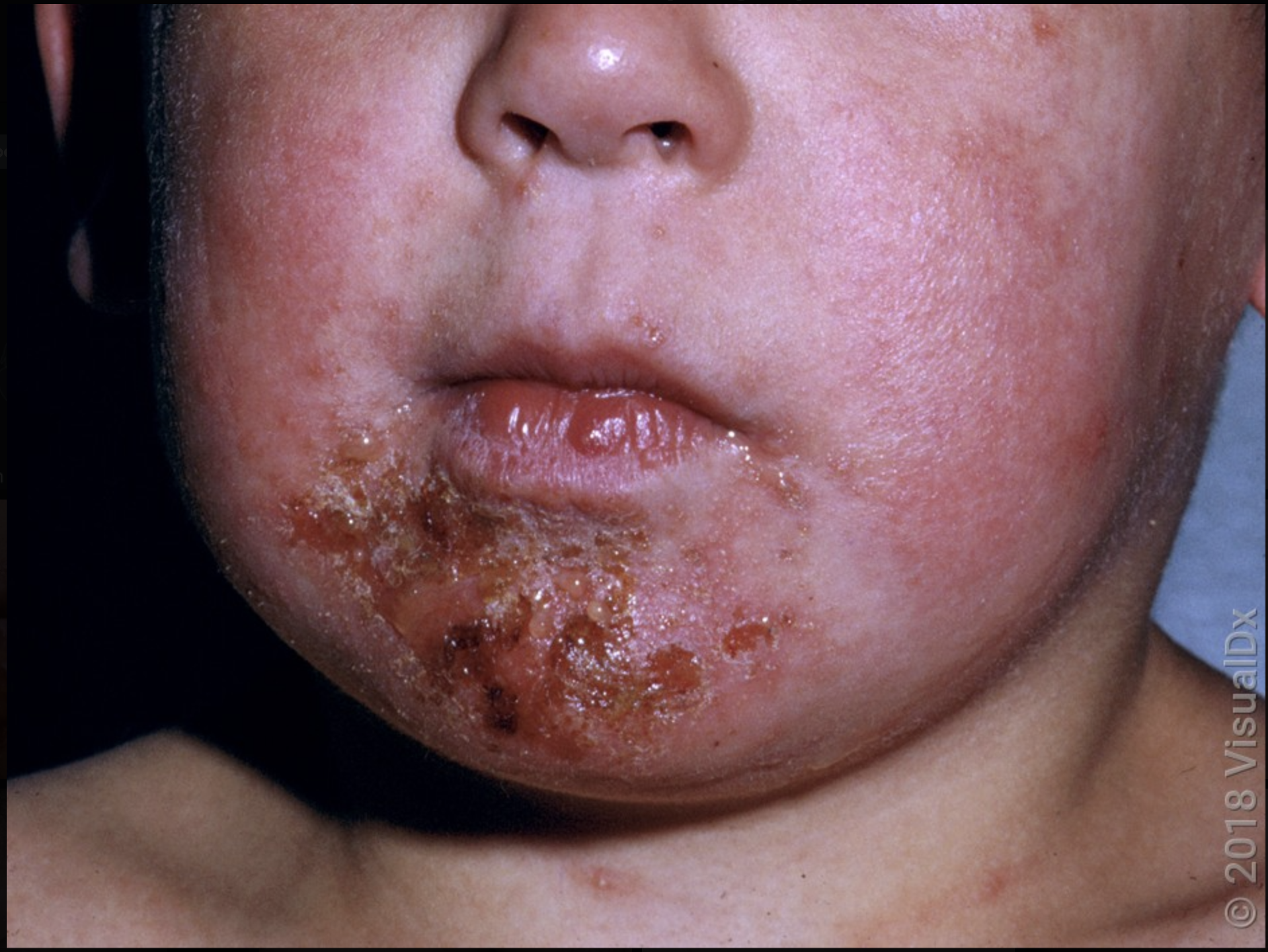

   1. Psoriasis
   2. Acrodermatitis enteropathica
   3. Tinea barbae
   4. Impetigo
   5. Eczema herpeticum

**Correct answer:** d. Impetigo

1.
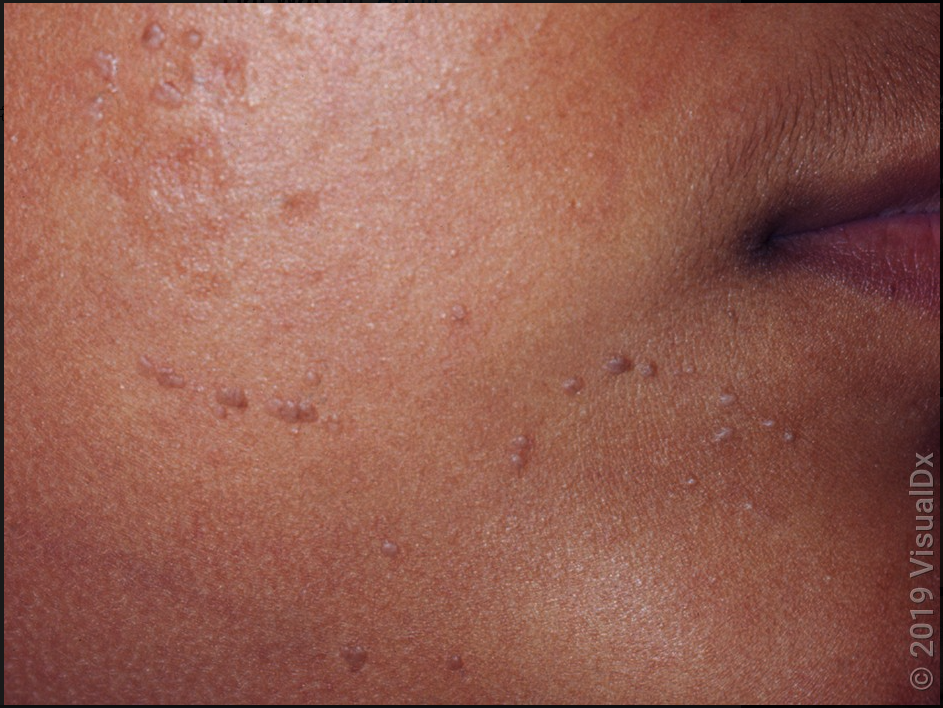

   1. Acrochordon
   2. Verruca vulgaris
   3. Seborrheic keratosis
   4. Inflammatory linear verrucous epidermal nevus
   5. Nevus sebaceous

**Correct answer:** b. Verruca vulgaris

1.
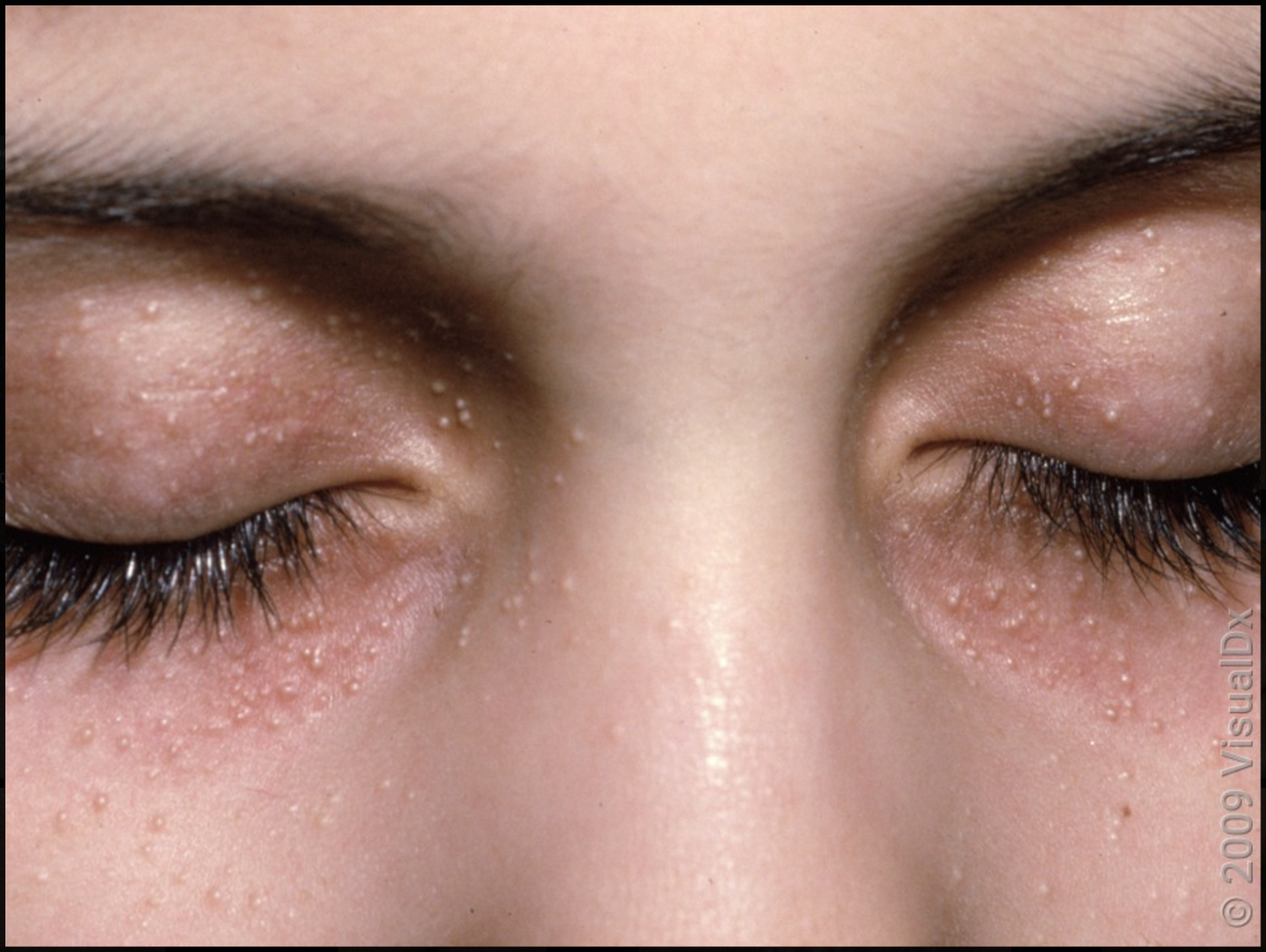

   1. Milia
   2. Syringoma
   3. Sebaceous hyperplasia
   4. Periorificial dermatitis
   5. Molluscum contagiosum

**Correct answer:** a. Milia

1.
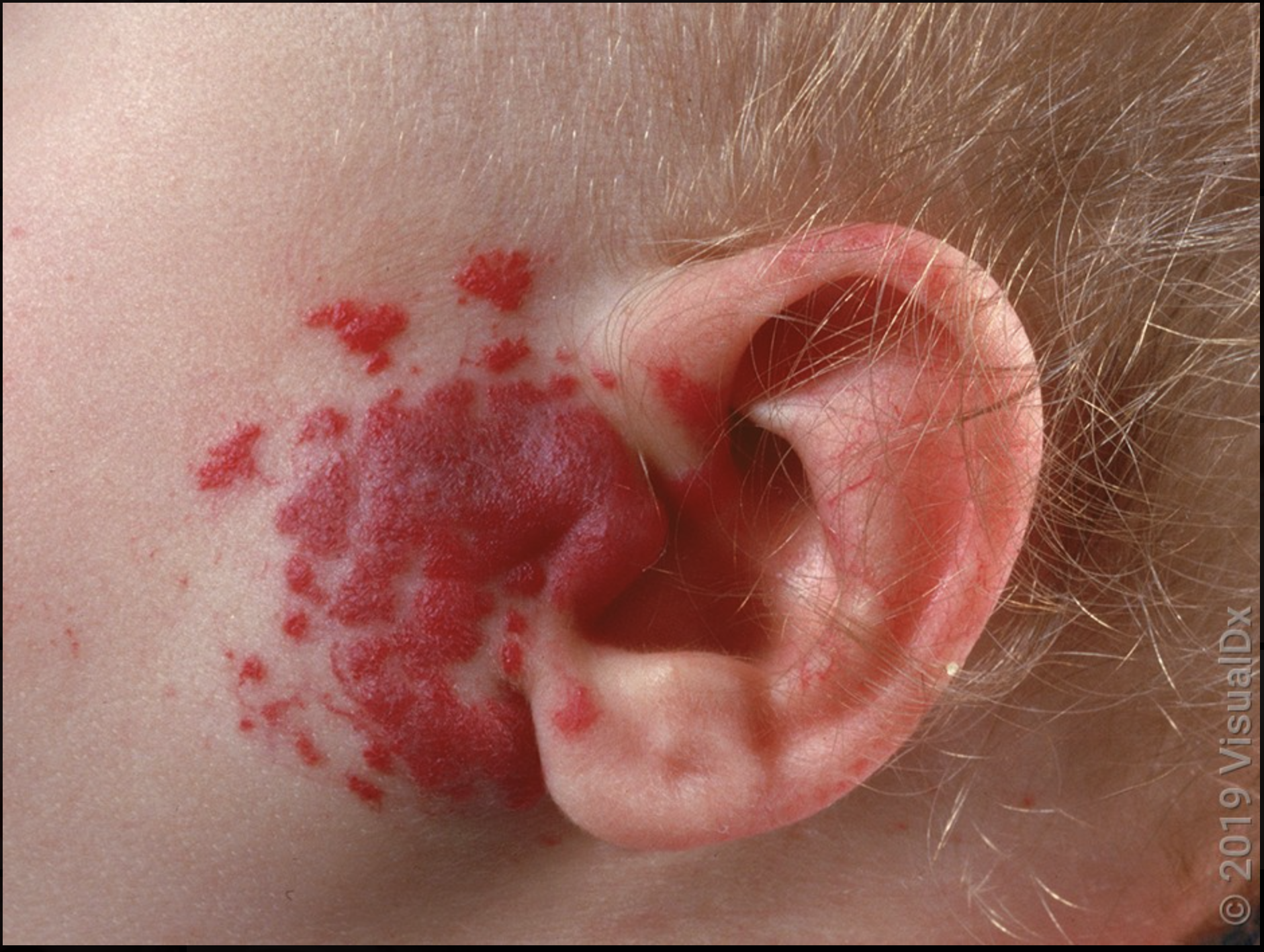

   1. Capillary malformation
   2. Nevus simplex
   3. Infantile hemangioma
   4. Pyogenic granuloma
   5. Tufted angioma

**Correct answer:** c. Infantile hemangioma
